# Binary DNA Nanostructures for Data Encryption

**DOI:** 10.1371/journal.pone.0044212

**Published:** 2012-09-11

**Authors:** Ken Halvorsen, Wesley P. Wong

**Affiliations:** 1 Immune Disease Institute/Program in Cellular and Molecular Medicine, Harvard Medical School and Children’s Hospital Boston, Boston, Massachusetts, United States of America; 2 Department of Biological Chemistry and Molecular Pharmacology, Harvard Medical School, Boston, Massachusetts, United States of America; Northeastern University, United States of America

## Abstract

We present a simple and secure system for encrypting and decrypting information using DNA self-assembly. Binary data is encoded in the geometry of DNA nanostructures with two distinct conformations. Removing or leaving out a single component reduces these structures to an encrypted solution of ssDNA, whereas adding back this missing “decryption key” causes the spontaneous formation of the message through self-assembly, enabling rapid read out via gel electrophoresis. Applications include authentication, secure messaging, and barcoding.

## Introduction

As the blueprint for all living things, DNA has a remarkable ability to robustly store and relay information. Two salient features of DNA make this possible: its modular construction from four distinct bases (A, C, G, T) whose sequence determines the genetic code, and the specific base pairing between complementary bases that enables hybridization into a double-stranded complex. These features of DNA have been exploited to perform computations and process information [1]–[6], including the hiding and encryption of secret messages [7]–[10]. Not only has DNA formed a foundation for the field of biomolecular computing, but the robustness of DNA-base pairing has also led to its use as a *programmable* structural material to construct objects with nanoscale features [11]. Recently, the accessibility and versatility of DNA nanotechnology has been remarkably increased with an approach referred to as DNA origami [12]. With a carefully designed collection of oligonucleotides, DNA can self-assemble into 2D and 3D shapes of stunning complexity [12]–[14], some of which incorporate sensing and actuation [15], [16]. Combining the concepts of DNA as an information processing molecule and as a structural material, we have developed a simple and powerful approach for encoding and encrypting information.

**Figure 1:**
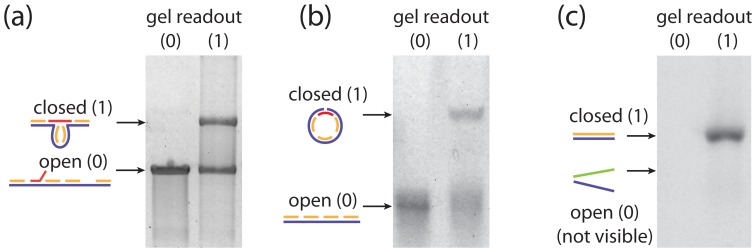
DNA as a binary switch. The conformations of two-state DNA nanostructures can represent bits through open or closed states, representing 0 and 1 respectively. We demonstrate this concept with three different implementations: a) a self-assembled construct with an addressable loop closure as described previously [17], b) a switchable circular/linear construct, and c) a double-stranded/single-stranded segment.

In previous work, we used DNA origami methods to construct a nanoscale mechanical switch [17], which we used to study the force-dependence of molecular interactions at the single-molecule level. This switch could be in one of two states, looped or unlooped, and we observed that these conformational differences were clearly resolvable in an agarose gel (Figure 1a). Thus, while other schemes for representing binary data using DNA have been presented [8], [18], [19], here we focus on using the geometric conformation of DNA nanostructures to encode binary values, due to the ease of both encoding and decoding information using this approach. We have made three distinct realizations of this concept, as demonstrated in Figure 1. These nanoscale structures can switch between two distinct states, acting as a “mechanical bit” to enable the storage and processing of information (analogous to mechanical relays in the earliest digital computers). These mechanical bits can be prepared in either state (0 or 1), and transitions between these states could be controlled using chemical or physical means (e.g. by changing the presence or absence of a critical molecular component, by varying the temperature, by interactions with light [20], or by applying mechanical force [17], [21]). Importantly, the state of these bits can be read out in minutes using gel electrophoresis, or faster using single-molecule imaging and manipulation techniques, and multiple bits can be represented with different lengths of DNA to facilitate multiplexed information processing and readout.

Each mechanical bit is formed via DNA self-assembly, and can be encrypted by omitting a critical component of its structure (e.g. a key single-stranded DNA molecule) that reduces it to an unstructured mixture of oligonucleotides. Messages encrypted as a collection of such bits are difficult to decipher since the 0 bits and 1 bits are nearly indistinguishable mixtures of oligonucleotides that are identical in all but sequence. On the other hand, decryption with a key is very easy–simply adding the missing component triggers the self-assembly of these nanoscale mechanical bits into their unencrypted forms. The separation of each mechanical bit into two parts or two “keys” forms an asymmetric encryption system. This system has the “public key” property if the key is distributed physically, since one key cannot be readily determined from the other without knowledge of the sequence. Furthermore, suitable countermeasures, such as adding “distractor” oligos to the physical encryption key to obscure information, can make the decryption sequence difficult to obtain.

**Figure 2:**
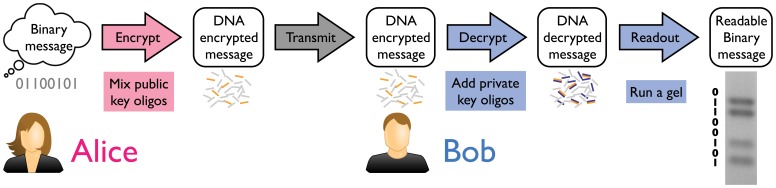
Conceptualization of DNA encryption and decryption. Alice prepares her message by mixing together oligos that correspond to either a binary 0 or 1 for each bit. This mixture is sent to Bob through a public channel, who decrypts the message by adding the DNA decryption key. This causes the message to self-assemble, enabling rapid read out by gel electrophoresis.

As an example, let us consider how Alice could send an encrypted message to Bob (Figure 2) using the linear binary switch shown in Figure 1C. Suppose she would like to send a three bit message, such as “101”. First, Bob must generate the appropriate DNA encryption and decryption keys for each bit. To distinguish between bits, he chooses each to be a different DNA length, (e.g 20 bases, 30 bases and 40 bases), and then generates 3 equal length oligos for each bit (A, A’, and B), two of which are complementary and hybridize together (A and A’) and one of which is inert (B) (see Materials and Methods for details on oligo design). Then Bob makes vials of A and B for each bit available, which represent the 1 and 0 values, respectively, while keeping the vials of oligo A’ private. Together, the vials of A and B oligos form the *encryption key*, which can be used by anyone to encrypt a message. To send a message to Bob, Alice would mix either A (for a 1) or B (for a 0) for each bit into a single vial and send this mixture to Bob over a public channel. At this point, only Bob can decrypt the message by mixing in the private *decryption key* (the set of A’ oligos) and running a gel–even Alice has no way to decrypt her own message once it’s been made.

## Results and Discussion

**Figure 3:**
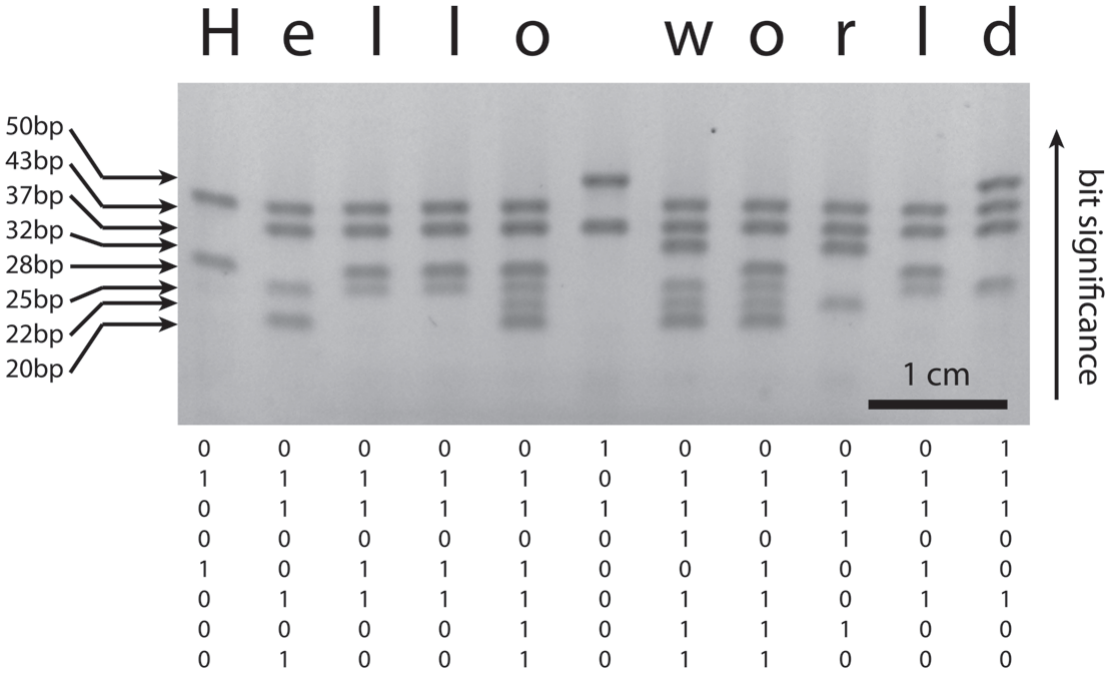
Decoding a binary message on a gel. Each lane of the gel contains a mixture of oligonucleotides which together form an 11 byte binary ASCII message which reads “Hello world”. The bit strings are read from top to bottom with the most significant bit being the largest DNA segment. Presence of a band indicates a binary 1, while absence of a band indicates a binary 0. All lanes have the same amount of DNA present, but only the double stranded pieces dye.

We experimentally demonstrated this encryption scheme for an 8-bit encoding by using 8 different lengths of DNA strands to represent the individual bits. We encoded a plaintext message using an 8-bit binary ASCII encoding with the 8th bit as an even parity bit, and encrypted the message into an ordered sequence of DNA mixtures, one for each letter. These mixtures were then decrypted by mixing them with the private key oligos and read out by immediately running an agarose gel. Reading each gel lane from top to bottom, the decrypted message “Hello world” becomes unambiguously clear (Figure 3).

One of the most intriguing aspects of encrypting messages with DNA as described is the difficulty for an interceptor or attacker to decrypt it. At a minimum, decryption attempts require possession of the physical message as well as technical skill, laboratory equipment, and time. Since the message is encrypted physically, the transmission of data can be well controlled, and even copying the encrypted message poses a significant technical challenge. Unlike encryption schemes that rely on mathematical algorithms, our biochemical based encryption is not directly vulnerable to increasing computational power. With physical decryption, the number of decryption attempts is limited by the availability of physical material comprising the message, which is depleted with each attempt. In fact, the message could theoretically be reduced to enable only a single decryption attempt.

Attempts to crack the message without the decryption key would be difficult, especially for an attacker limited to the same resources as the intended recipient (i.e. electrophoresis and mixing equipment). For example, a näive brute force attack to find the decryption key would require the physical generation and testing of an astronomical number of possible keys, with 4*^N^* distinct possibilities, or 10^155^ for our simple 8-bit encoding scheme (and an approximately 1 in 10^63^ chance of guessing a decryption key if we allow for 25% mismatched bases [8], [22]). However, aside from the correct decryption key, there could also exist a pseudo key or set of pseudo keys that could allow an attacker to distinguish between individual mixtures, providing a toehold for unauthorized decryption using language statistics on a large set of messages. Additionally, if the attacker has access to more sophisticated approaches, including sequencing techniques, DNA profiling with microarrays, or the ability to make libraries of oligos to test multiple keys at a time, then the security will be reduced. Fortunately, many of these approaches can be impeded by implementing the appropriate countermeasures, as described below. In addition, it is interesting to note that many of the potential ways to crack the code may involve significant technical expertise, expensive laboratory equipment, or time consuming processes, giving rise to a large asymmetry in effort compared to encrypting and decrypting the message with the proper keys.

To maximize encryption security, a variety of countermeasures can be used that comprise a two pronged approach: 1) limiting access to physical information about the message (e.g. the oligo sequences), and 2) making this physical information difficult to decipher. One simple countermeasure is to limit the physical amount of material comprising the message, as mentioned above. Additionally, we can impede chemical analysis by modifying the ends of the oligos to prevent the chemical conjugation required for some sequencing and profiling techniques. One important countermeasure against unauthorized decryption that falls into both categories is the addition of noise by mixing “distractor strands” into the physical message (described in detail elsewhere [8], [23]). Adding oligonucleotides with a similar length and composition as the message strands but with different sequences increases the time and effort required to obtain the sequences of the oligonucleotides within the encrypted mixture, which is already a challenge due to their short lengths [24]–[26]. Also, distractor strands make deciphering the message more difficult since the true message can be obscured by noise designed to like the signal, or vice versa [23]. For a public key system, special care should be taken in designing the distractor strands to ensure that the two values for each bit are difficult to distinguish using a pseudo key or other biochemical techniques (e.g. mass spectrometry). For a private key system, distractor strands can be very effective at deterring statistical analysis, as each occurrence of a given letter could be mixed with a different set of distractor strands. In fact, false messages could be encoded into the mixture with distractor strands that represent “false encryption keys”, and the attacker would have little way to identify the true message without knowing the specific decryption key sequence. Alternatively, to make the signal look more like the noise, DNA structures could be pre-encrypted with standard computer encryption algorithms. Furthermore, cracking the code becomes increasingly challenging the more distractor strands are added, and the more bits per message are encoded.

While our demonstration showed 1 byte (8 bits) of data storage per mixture (and per gel lane), the amount of data stored and read out could be readily increased. If DNA lengths were optimized to be evenly spaced on the gel, a resolution of 1 mm would give 10 bits/cm of gel length, corresponding to roughly 8 bytes of data for a single short gel lane, 10–20 bytes for longer gels, and up to 125 bytes (∼1000 bits) for more elaborate sequencing gels with base pair resolution [27]. Expanding to a few bytes could enable the transmission of entire words or short messages in a single mixture, especially if a more efficient character encoding scheme were used (e.g. the 5 bit Baudot code), or a word-based encoding scheme (e.g. 10 bytes could encode eight words of a 1000 word vocabulary). In addition, data density could be dramatically increased by combining multiple messages within a single mixture, with each message associated with a different decryption key. We note that these approaches will improve security by making statistical analysis of the message difficult.

In summary, we have developed a novel technique for encoding binary information in two-state DNA nanostructures, for encrypting and decrypting this information using self-assembly, and for rapidly reading this information back out with gel electrophoresis. As the state of such nanostructures can be used to report molecular events (such as the rupture of intermolecular bonds [17]), additional applications of this work beyond information security include the characterization of molecular interactions with a multiplexed gel readout. More directly, this approach provides a relatively simple and inexpensive way to send secure, and potentially hidden, messages over a public channel. Unlike similar DNA encryption methods that require specialized laboratory work (e.g. PCR, sequencing, cloning) that can take hours to days [7]–[10], our technique enables encrypting and decrypting messages in just minutes, requiring as little as disposable droppers and a no-prep bufferless (even handheld) gel system (e.g. Invitrogen E-gel systems). This convenience and simplicity also opens the technology to other applications such as authentication and barcoding. For example, increasing storage capacity to 40 bits would enable storage of the ubiquitous 12 digit Universal Product Code (UPC). Several methods have been demonstrated for storing printed DNA on paper [28], [29], which combined with our quick readout method could enable a new level of security in product identification. In fact, tagging with DNA inks has recently found commercial use (DNA Technologies, Halifax, Canada) in anti-counterfeiting efforts for a variety of products including sports memorabilia, artwork, pharmaceuticals, and luxury goods. As previously proposed, DNA barcodes may also find use in labeling of liquids such as paint and oil, or possibly even food [8]. As another example, it could provide a simple and inexpensive way for pharmaceutical companies to discretely label drugs with production or expiration information, even at the level of edible encrypted barcodes on individual tablets. This is particularly timely in light of recently introduced government mandates (e.g. California E-Pedigree Law) to serialize pharmaceuticals and to ultimately “track and trace” them, since our method offers a way to identify and authenticate drugs, reducing theft and counterfeiting. One could also envision personal identification cards (e.g. driver’s license, passports) being printed with DNA markers on them as an additional prevention against fraud or identity theft, or even using one’s own genomic DNA as an authentication key.

## Materials and Methods

We designed and purchased oligos (Bioneer, Inc.) to represent 8 different bits, which would be about evenly spaced on a 4% agarose gel. The lengths we chose were 20 nt, 22 nt, 25 nt, 28 nt, 32 nt, 37 nt, 43 nt, and 50 nt. For each length, 3 oligos were purchased: a randomly generated sequence, it’s complementary strand, and a random set with arbitrary bases. We denote these as set A, A’, and B respectively.

To encode messages, we first converted our plain text message “Hello world” into binary code using 8 bit ASCII character encoding with the 8th bit as an even parity bit for error checking. Since we have 8 bits total, each letter was prepared using a mixture of A and B oligos to represent the 0 s and 1 s. As an example, the “H” in “Hello world” has an 8 bit binary representation of 01001000. The least significant bit is encoded in the smallest (20 nt) oligo, which we will denote oligo 1. To encode the “H”, we mix oligos 4 and 7 from set A with oligos 1, 2, 3, 5, 6, and 8 from set B.

For decoding, the encoded message mixtures were individually mixed with the entire set A’ of oligos in a buffer solution (1× Buffer 4, New England Biolabs) and loaded into a gel (within minutes). We ran an automated precast 4% agarose gel (E-gel, Invitrogen) containing a proprietary dye (with characteristics remarkably similar to Sybr gold) for 15 minutes and took a picture immediately after. The binary representation of each gel lane could be read directly from top to bottom.
